# CONDOR: a database resource of developmentally associated conserved non-coding elements

**DOI:** 10.1186/1471-213X-7-100

**Published:** 2007-08-30

**Authors:** Adam Woolfe, Debbie K Goode, Julie Cooke, Heather Callaway, Sarah Smith, Phil Snell, Gayle K McEwen, Greg Elgar

**Affiliations:** 1 School of Biological Sciences, Queen Mary, University of London, Mile End Road, London E1 4NS, UK; 2 Genomic Functional Analysis Section, National Human Genome Research Institute, National Institutes of Health, Rockville, MD 20870, USA

## Abstract

**Background:**

Comparative genomics is currently one of the most popular approaches to study the regulatory architecture of vertebrate genomes. Fish-mammal genomic comparisons have proved powerful in identifying conserved non-coding elements likely to be distal *cis-*regulatory modules such as enhancers, silencers or insulators that control the expression of genes involved in the regulation of early development. The scientific community is showing increasing interest in characterizing the function, evolution and language of these sequences. Despite this, there remains little in the way of user-friendly access to a large dataset of such elements in conjunction with the analysis and the visualization tools needed to study them.

**Description:**

Here we present CONDOR (COnserved Non-coDing Orthologous Regions) available at: . In an interactive and intuitive way the website displays data on > 6800 non-coding elements associated with over 120 early developmental genes and conserved across vertebrates. The database regularly incorporates results of ongoing *in vivo *zebrafish enhancer assays of the CNEs carried out in-house, which currently number ~100. Included and highlighted within this set are elements derived from duplication events both at the origin of vertebrates and more recently in the teleost lineage, thus providing valuable data for studying the divergence of regulatory roles between paralogs. CONDOR therefore provides a number of tools and facilities to allow scientists to progress in their own studies on the function and evolution of developmental *cis*-regulation.

**Conclusion:**

By providing access to data with an approachable graphics interface, the CONDOR database presents a rich resource for further studies into the regulation and evolution of genes involved in early development.

## Background

In recent years, although great advances have been made in our ability to annotate gene sequences, the annotation of sequence elements that control complex gene expression is lagging far behind. With exons accounting for only a tiny proportion of the genome (< 1.5%) there remains a vast amount of unannotated sequence, containing an unknown number of functional elements. In particular, the annotation of the complex network of *cis*-regulatory modules (CRMs), such as enhancers and silencers, that coordinate the precise spatial and temporal expression of genes during development presents one of the biggest challenges of the post-genomic era. This is predominantly because we know so little about their language, mode of action and origin, and traditional ways of identifying them are difficult and laborious. The burgeoning field of evolutionary developmental biology (termed evo-devo) is increasingly shifting its emphasis towards the importance of gene regulatory networks in the evolution of developmental pathways [1]. Within evo-devo there is therefore growing interest in the identification and characterization of the CRMs that control these networks. With the completion of an increasing number of vertebrate genomes the correlation between sequence conservation and biological function provides a powerful resource for the discovery of functional elements. Indeed a number of recent large-scale comparative studies have resulted in the identification of thousands of evolutionary constrained elements in the human genome (e.g. [2-5]). These conserved non-coding sequences represent a diverse set of functional elements, a proportion of which are likely to act as CRMs.

The confidence with which putative functional non-coding elements can be identified depends on the evolutionary distance between the species selected, and a balance must be found between eliminating background noise and retaining sufficient sequence similarity for detection [6]. The inclusion of fish genomes in comparative analysis has proved a highly successful approach to filter and prioritize non-coding sequences most likely to be functional.

The large evolutionary distance between fish and mammals means that even the slowest evolving DNA has diverged sufficiently to significantly improve the signal to noise ratio in genomic alignments. In particular the genome of the pufferfish, *Fugu rubripes*, with its highly compact non-coding regions, has been commonly used as a model genome to increase the efficacy of detecting putative functional sequences. The similarities between mammals and *Fugu *are recognisable despite their large evolutionary divergence. Both vertebrates, they share not only the defining characteristic of a backbone, but also many basic anatomical and physiological similarities and patterns of development. The strong parallels in early developmental patterning are likely to be under control of a common core set of genes and regulatory elements. Significantly, a strong spatial association between highly conserved non-coding elements (CNEs) and genes involved in transcriptional regulation and/or development (termed *trans-dev *genes) has been described in many studies [3,4,7-9]. Functional testing has shown that conserved non-coding elements identified using very strict conservation criteria in mammals (e.g. ultraconserved elements [3]) or using evolutionary distant species [4,10] are considerably enriched for enhancer sequences. In both mouse [11] and zebrafish [4], a large proportion of the elements tested *in-vivo *function reproducibly to up-regulate tissue-specific expression of reporter genes during development.

With an increasing supply of functional data, we have created CONDOR (Conserved Non-coDing Orthologous Regions), a database resource for the study of evolutionary conserved *cis*-regulation in early developmental genes. Here, such elements are rigorously defined by optimal alignment strategies for sequences as divergent as fish and tetrapods, ensuring that sequences are derived from orthologous regions. In addition, we cater for the growing number of researchers in evo-devo interested in sets of experimentally verified developmental enhancers. Thus we couple both bioinformatics and functional data in a visual and searchable form.

## Construction and content

The relational database structure can be divided conceptually into two main parts, representing the two main sources of data stored within it. The first relates to the computationally derived dataset from *Fugu*-mammal multiple alignments, in particular the CNEs identified from them. The second stores functional (lab-based) data from *in vivo *enhancer assays carried out in-house. While these two datasets are integrated in the database their derivation will be outlined separately.

### Computational dataset

**Figure 1:**
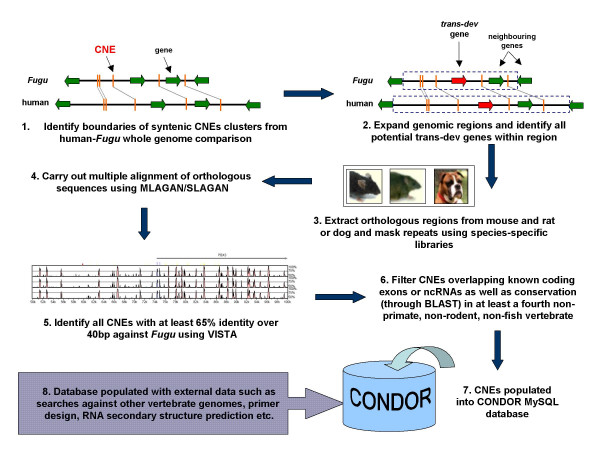
**Methodology for alignment and identification of CNEs between *Fugu *and mammalian orthologous genomic sequences**. Boundaries of CNE-containing regions are identified through a CNE synteny map created using a stringent whole genome comparison of the non-coding portions of the human and *Fugu *genomes [4]. The genomic regions to be aligned are then expanded past the map boundaries up to the next nearest known genes. *Trans-dev *genes in the region are determined using appropriate GO ontologies and/or InterPro domains. Orthologous sequence corresponding to this expanded region in human is then extracted from mouse, rat and/or dog genomes. Sequences are masked for repeats and are aligned using MLAGAN (identifying sequences conserved in the same order along the sequence) and SLAGAN (to identify conserved elements that have undergone rearrangement in one or more lineages). CNEs are identified using VISTA and filtered to exclude any that overlap known coding exons or ncRNAs. As an added stringency filter, only those CNEs that are conserved in at last four divergent vertebrate genomes (including *Fugu*) are retained to avoid spurious matches.

**Figure 2:**
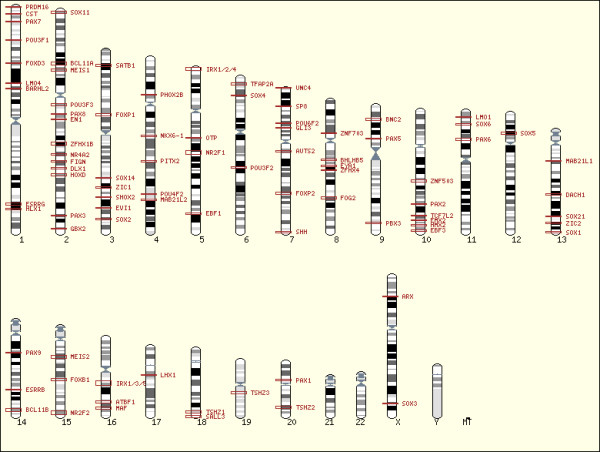
**Chromosomal locations of developmental gene regions currently covered in CONDOR**. Red outlined boxes represent regions across which CNEs are distributed, and are proportional to the size of the region. The reference *trans-dev *gene to which the region is associated is marked next to the box. In most cases this is the only *trans-dev *gene in the vicinity although in a number of cases CNEs are interspersed within clusters of related *trans-dev *genes (e.g. the *HOXD *cluster) or within clusters of unrelated *trans-dev *genes (e.g. the PAX1 region which contains the *trans-dev *genes *PAX1*, *NKX2.2*, *NKX2.8 *and *FOXA2*). CNE regions in CONDOR are found on all chromosomes except 21, 22 and Y. Figure created using Ensembl Karyoview [25].

The core of the CONDOR database is made up of a set of more than 6800 CNEs identified through a combination of sensitive multiple and multi-pairwise alignments of orthologous regions between a baseline teleost *Fugu *genome and four mammalian genomes including human, mouse, rat and dog. The full methodology used to identify CNEs can be seen in Figure 1. To ensure all alignments were carried out on contiguous orthologous sequence, it was necessary in some cases to bridge gaps and extend *Fugu *scaffolds using additional assembly data (such as BAC ends and sequence from older assemblies) from the Fugu Information Network [12]. The alignments are targeted to regions previously identified genome-wide as containing syntenic clusters of CNEs between the *Fugu *and human genomes [4]. These clusters are almost exclusively located in the vicinity one or more *trans-dev *genes. The CONDOR database is therefore designed around these distinct regions. Multiple alignments are more sensitive and better suited for identifying conservation between highly diverged sequences such as fish and mammals than more commonly used pairwise alignments [13] and were carried out using the MLAGAN alignment toolkit [14]. Currently the database covers 100 clusters of CNEs (including ten that are duplicated in *Fugu*) encompassing 129 *trans-dev *genes conserved in synteny with these clusters in both fish and mammals. The chromosomal locations of developmental gene regions currently in the database can be seen in Figure 2. The CNEs are carefully filtered to exclude all elements that overlap known coding exons and non-coding RNAs as well as repetitive and low-complexity sequences. CNEs falling within UTRs are retained, as it is not currently known whether these function in a pre or post-transcriptional manner.

**Figure 3:**
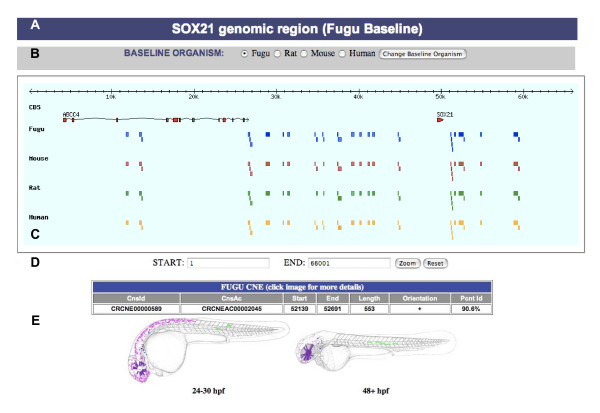
**Example of the "CONDOR View" graphical browser for CNEs in the vicinity of the SOX21 gene in *Fugu***. Letters indicate the following features. (A) Title bar shows the reference *trans-dev *gene and the current baseline organism. (B) Option allowing users to change the baseline organism co-ordinate system to which CNEs are mapped on the graphic. (C) Clickable graphic/image map representing all CNEs across the baseline sequence. Top bar represents the length of the sequence in kilobases (here ~66 Kb). The CDS track shows gene structures (exons indicated as red boxes) with the end arrow indicating the strand direction of the gene. The four organism tracks represent the organism sequences used in the initial MLAGAN/SLAGAN alignments. CNEs are positioned according to the baseline co-ordinate system and drawn as boxes with lengths relative to their size in bps. CNEs are 'bumped' onto lower lines if located too close to another CNE for them to be differentiated on a single line. While all CNEs are mapped to the baseline sequence, boxes are drawn in the other organisms if they are conserved to that CNE in the MLAGAN alignment. Moving the mouse over a specific CNE in one of the organism tracks brings up summary data on that CNE in a table below the browser (E). If the CNE has functional annotation (as shown) a composite schematic of GFP expression is displayed underneath. Clicking on the image map opens up a new web page with detailed information on the CNE. (D) Users can zoom in or out of the image map to get a clearer view of specific sets of CNEs (useful in larger regions).

Following initial identification, CNEs are searched for further conservation in other available vertebrate genomes that currently include seven mammals, one bird, one amphibian and four teleosts. It is clear from a number of recent studies that distal *cis*-regulatory elements can exert their regulatory influence large distances from their target gene [15], from within the introns of neighbouring genes and across intervening genes [16]. The orthologous regions used for alignments are not limited to the intergenic space surrounding the *trans-dev *gene(s) but rather by the initial whole-genome CNE synteny map resulting in the identification of CNEs that may be located large distances from the *trans-dev *gene or within the introns of neighbouring genes (e.g. Figure 3). As these CNEs have retained conserved syntenic order with the *trans-dev *gene within orthologous regions of both mammalian and fish genomes (despite these lineages having undergone significant genomic rearrangement since divergence [17]) they are still likely to be associated with the most proximate developmental gene(s) [18]. CNEs that have undergone rearrangement (such as inversions and transpositions) in one or more lineages are identified from the same orthologous sequences using Shuffle-LAGAN, filtered in the same way and integrated into the final set [19]. The emphasis in CONDOR of utilising alignments of strictly orthologous sequence in addition to relaxed conservation criteria appropriate for fish-mammal distances increases the ability to identify a larger number of CNEs whilst decreasing the probability of getting spurious matches. Indeed CNEs identified in this way have a number of advantages over automated whole genome alignment methods employed in similar comparative genomic datasets that are highly human-centric such as the VISTA Enhancer Browser [20]. The first is the guarantee that CNEs in CONDOR are indeed conserved across vertebrates and their derivation is truly orthologous and not a potentially spurious non-orthologous match. The second is that the ability to rapidly map and survey CNEs in their proper genomic context in both fish and mammals acts as a useful filter in assigning likely target genes, that would not be possible looking solely at the mammalian context [21]. The high level of genome rearrangement and gene loss since the tetrapod/teleost split [17] means in many cases genes that are interspersed or located close to CNEs in mammalian genomes are no longer present as orthologs when viewing the same CNE region in the *Fugu *genome, leaving only the remaining *trans-dev *genes conserved in both genomes as potential targets [21].

Numbers of CNEs in each region in CONDOR vary considerably, ranging from two in the *PAX8 *region to 342 in the *IRX3/5/6-SALL1 *region, possibly reflecting differences in regulatory complexity and the extent to which regulation has been conserved between fish and mammals as well as the possible presence of overlapping regulatory domains. CNEs in CONDOR range in length from 21–864 bp (mean = 117 bp) and cover some 750 Kb of human sequence, or around 0.05% of the non-repetitive genome. Therefore, although this set represents a tiny fraction of the ~5% of the human genome thought to be under selective constraint [22] it is proven to be of the highest regulatory potential in development [4,23,11].

It is important for future analyses that published sets of tested CRMs identified through species comparisons can in some way be traced back to a sequence repository and thus properly defined, similar to the definition of sequences in protein or transcript databases. Commonly in currently published work, functionally characterized conserved non-coding sequences are referred to by some arbitrary name and by their location in the genome or the distance from the start of a gene. This often changes with the release of updated genome assemblies and makes it difficult for other researchers to track the sequence back from the literature. In response, CONDOR is the first database to use specific identifiers for conserved non-coding elements in each genome, independent of their current location in a genome assembly. Unique identifiers are used both to refer to a CNE orthologous unit (akin to a cluster of orthologous genes), e.g. CRCNE00000054, which is useful in defining a CNE in any vertebrate genome to which it is conserved, as well as individual sequences within the CNE, e.g. CRCNEAC00000172 (in human), from sequences used to define the element in the original multiple alignment (i.e. human, mouse, rat, dog and *Fugu*). Specific identifiers are only assigned to sequences in these species (as opposed to any of the other vertebrate genome in which a CNE is present), as they are only sequences that can be strictly guaranteed to derive from orthologous regions.

### *In vivo *enhancer assay dataset

The CONDOR database also serves as a repository of CNEs experimentally tested *in vivo *for enhancer activity, with around 100 elements currently tested. Details of this *in vivo *assay using the zebrafish model *Danio rerio *have been published previously[4]. Briefly, as candidate enhancer elements, CNEs are PCR amplified using primers designed as close to the conserved sequence as possible. This guarantees that as far as possible any activity from the assay derives directly from the conserved sequence and not from undetected regulatory signals in non-conserved flanking sequence. Other enhancer assays (e.g. [11] and [24]) often include large regions of sequence that flank the conserved element, making it increasingly difficult to narrow down the sequences responsible for enhancer activity. The PCR product is co-injected together with a GFP reporter construct under the control of a human β-globin minimal promoter into zebrafish embryos before the eight cell stage. Embryos are generally screened for GFP expression 24–30 hours post fertilization (hpf) and again at 48–54 hpf. It is not thought that the injected DNA is integrated into the genome leading to mosaic expression. Therefore for each assay, we generate GFP expression data from a minimum of 25 embryos in order to obtain an overall expression profile. Using graphics software, this profile is depicted schematically as color-coded cells drawn onto an overlay of a camera-lucida drawing of either a 24–30 hpf or 48–54 hpf embryo. The color-coding relates to nine broad expression domains (e.g. central nervous system, sensory organs etc) that can be visualized as separate schematics. These are further subdivided into one or more localized expression domains (e.g. forebrain, midbrain and hindbrain) and the proportion of embryos with expression in each sub-domain is shown as a bar chart.

The transparency and rapid development of zebrafish renders it a popular model organism for developmental biology. Unlike the mouse model, large numbers of embryos can be screened and GFP expression can be readily monitored in a live embryo. Also mouse embryos are opaque, and so without dissection, low levels of expression may remain undetected. Since complementary results can be obtained when assaying human-fish conserved elements in either model system [25], data from our *in vivo *assay represents a valuable foundation for prioritizing more focused studies in a mammalian system. As with any assay system there are a number of caveats associated with interpreting results. Elements are tested out of their genomic context and may not produce the same patterns of expression as they would in the genome, possibly due to structural or steric influences or interactions with other elements not present in the assay. In addition, CNEs that test 'negative' in our assay may act as enhancers in time points beyond the assay, or in rare cell types. Alternatively, they may be acting as another type of functional element such silencers, structural regulators or ncRNAs. Users should therefore take these caveats into consideration.

The number of tested elements in the database will continue to grow in the future as in-house gene-centric studies continue and new techniques are employed and developed (such as a Tol2 integration [26] and recombineering), although no systematic assaying of all elements in the database is currently planned. The number of labs using zebrafish embryos to assay developmentally associated CNEs for enhancer activity is increasing rapidly. We therefore invite scientists using this assay to contribute their annotations from such studies (following publication) to CONDOR to make the database a community resource and allow those results to become more widely available and distributed.

## Utility and discussion

Both comparative genomic data and *in vivo *results are publicly available as a web-based resource. The website is designed to make the interrogation and visualisation of this data as user friendly and intuitive as possible, catering for both lab-based investigators interested in biological function and bioinformatics researchers interested in sequences datasets of high regulatory potential. The web site therefore provides a number of approaches by which the data can be accessed, visualised and downloaded.

### Interrogation and visualization of CNEs by genomic region

The central search page allows CNE-containing gene regions to be located from a pull-down list or searched more generally through a text search by gene name, protein accession number, GO term etc for any genes contained within these regions. Once located, CNEs within a gene region can be visualized via an interactive graphical browser (termed 'CONDOR View') or in a tabular form (termed 'Text View'). Each gene region analysed extends as far as the shared synteny and gene order between fish and mammals, allowing for long-range *cis*-regulatory elements that may lie within or beyond neighbouring genes. These regions are represented in CONDOR View so that users can graphically view the positions of CNEs along a chosen baseline genome in relation to other vertebrate CNEs. The conservation or absence of CNEs in other genomes used in the initial multiple alignment are also indicated through separate tracks in the browser. An example of 'CONDOR View' for CNEs in the *SOX21 *region in *Fugu *can be seen in Figure 3. By mousing over a CNE, simple information such as position, length, conservation etc. is displayed and an overview of enhancer activity is also shown for elements that have been tested (Figure 3). Users can zoom in and out of the browser to view specific regions in more detail (particularly useful in very large regions spanning several megabases) as well as select only CNEs that have been tested for enhancer activity. Directly clicking on the CNE in the browser opens a separate webpage with detailed sequence, evolutionary and *in-vivo *assay data (if available).

**Figure 4:**
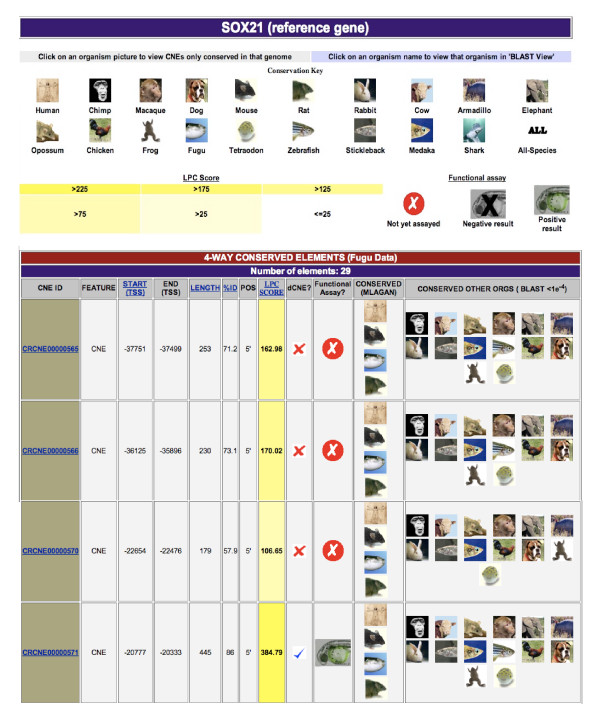
**Example of part of a "Text View" web results page for CNEs in the vicinity of the *SOX21 *gene in *Fugu***. The two top bars represent keys to data within the table relating to genomes in which CNEs are conserved, conservation scores and functional annotation. Links through names/pictures relating to all vertebrate genomes currently within CONDOR allow users to filter the CNEs by those conserved in a specific genome or to view the chromosomal locations of the CNEs in that genome. Main table shows data and features of each CNE in the region. The sequence data includes CNE identifier, location, position with relation to reference gene, length, conservation etc. CNE features shown also include the LPC score (a composite score that takes both the length and conservation of the element into consideration), whether a CNE is duplicated elsewhere in the genome (referred to as a dCNE or duplicated CNE [29]), whether the CNE has an *in vivo *annotation associated with it (and the general result of that annotation), and what vertebrate genomes it is conserved in.

'Text view' displays information on the CNEs in a tabular format providing additional data to that in 'CONDOR View', giving details of conservation in other vertebrate genomes (Figure 4) and highlighting CNEs that are duplicated elsewhere in the genome (see later section). Its main function is to allow users to prioritize a set of CNEs for further study by sorting them based on specific criteria such as length, conservation score or occurrence in any one of 19 vertebrate genomes currently in the database. For example, a user can choose to display zebrafish CNEs with the highest conservation scores within a specific gene region. In addition, the chromosomal or genome assembly positions of CNEs can be displayed for all 19 vertebrate genomes with external links to their position in the Ensembl Genome Browser. All assembly versions used in the database are promptly updated upon new releases.

The database is also available as a permanent DAS source within the most current human, mouse and rat assemblies at the Ensembl Genome Browser [27] so that any overlap between the CNEs and an increasing number of *in silico *and experimental annotations can be readily viewed. A similar track in the UCSC Genome Browser is also planned in the future.

### Searching CNEs by enhancer activity

**Figure 5:**
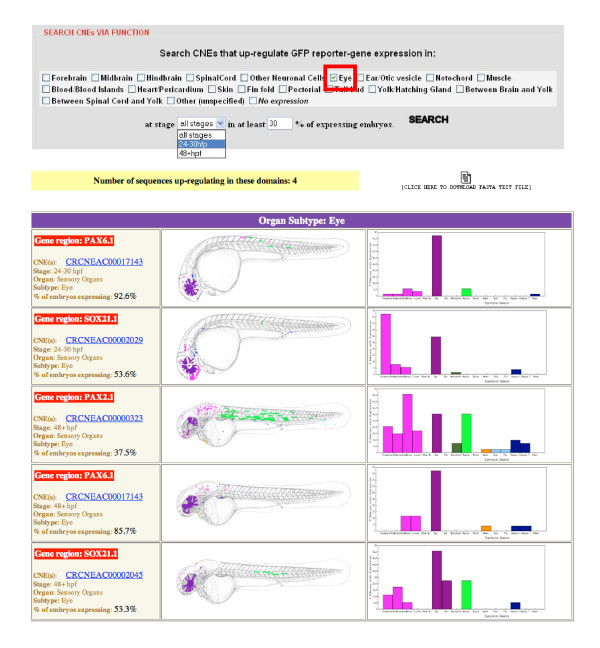
**Searching for CNEs through functional annotation.**  As well as viewing annotation for individual CNEs, sequences can be searched  for enhancer activity in one or more tissue types (in this example the eye)  and one or more developmental stages (24-30 and 48-54 hours post  fertilization). Parameters can also be changed to include only strong  expressers in the selected tissue type. A composite schematic of the  expression domains as well as a histogram of the proportion of embryos  expressing in each tissue type are shown for all annotations fulfilling the  search criteria. Hyperlinks are provided for each CNE to view annotations in  more detail. A multi-FASTA sequence file of the CNEs returned in the search  is provided to use in downstream analysis such as searches for  overrepresented words/conserved transcription factor binding sites etc  responsible for regulation to this tissue.

The vast majority of CNEs exhibit no sequence identity to any other sequence in the genome [4,28,29]. However, their seemingly unique nature stands in contrast to their apparent ability to drive expression to similar anatomical locations [4,11,23]. Therefore, while CNEs show little intra-genomic conservation at the sequence level, they are likely to share small common motifs representing protein-binding sites not readily detectable by sequence conservation algorithms. Indeed, a recent study of a broader set of conserved elements in mammals identified more than 200 overrepresented motifs, raising the possibility that specific motifs may be associated with specific classes of functional elements [30]. Clustering CNEs by their spatial and temporal specific enhancer activity to look for similar common motifs may therefore allow us to begin to discern the language of developmental enhancers. CONDOR provides a search facility that allows a user to search for tested CNEs driving expression in any of 19 anatomically distinct tissues and two developmental time points in zebrafish embryos. Figure 5 gives an example of search results for CNEs driving strong expression in the eye. A multi-FASTA file of all the resultant sequences is also returned to allow for further downstream analyses such as searching for common motifs or known transcription factor binding sites.

CONDOR will enable greater accessibility to ongoing functional data of *cis*-regulatory elements complementing and adding to the growing list of enhancers tested in the mouse model [20] with the added advantage of more developmental time points in zebrafish. As more annotated enhancers become available (in particular those confirmed by more than one assay system) statistical approaches aimed at deriving a regulatory language from this training set should become attainable.

### Studying duplicated elements and regulatory subfunctionalization

Whilst the vast majority of CNEs conserved across vertebrates are unique in the human genome [4,29] an interesting subset are part of multi-member families that share significant sequence similarity and derive from ancient whole-genome duplications at the origin of vertebrates [31]. These dCNEs (duplicated CNEs) are located in the vicinity of *trans-dev *paralogs and offer an important resource for studying *cis*-regulatory element evolution after duplication and how this influences paralogous gene function. It is worth noting that since CNEs can act over a long range, identifying their target gene can become complex when studying a region that contains more than one developmental gene. In the case of dCNEs, the retention of both element and gene after duplication, strongly implies association [32]. CONDOR highlights those CNEs that are duplicated (see Figure 4 as an example) and provides a tool to create alignments and locate genomic positions of all other members in any of the vertebrate genomes searched.

In addition, the CONDOR database is the first resource to make available data on CNEs identified from a number of teleost-specific duplicated regions within *Fugu *from alignments where both *Fugu *gene regions are compared independently to the same (ancestral) region in mammals. Recent comparative analysis of CNEs in these regions reveals assymetrical evolutionary rates and a pattern of retention and loss of CNEs between duplicated genes indicative of regulatory subfunctionalization [33]. To facilitate their study, CONDOR supplies an extra facility that allows the CNEs in these duplicated regions to be compared side-by-side highlighting those CNEs that have partitioned to a specific gene copy as well as those conserved to some degree in both copies.

### Additional tools and features

In addition to providing data on the locations and sequence information of each CNE, CONDOR also provides a number of other features to help study the function and evolution of these sequences:

• To facilitate the amplification of CNEs from a genome of choice for *in vivo *or *in vitro *analysis, a number of automatically generated PCR primer predictions using the EPrimer3 program [34] are provided for all CNEs. Alternatively, flanking sequence around the CNE of a user-defined size can be downloaded to allow manual primer design.

• Sensitive BLAST alignments of any CNE against a variety of nucleotide sequence databases can be displayed dynamically (where the user can choose the e-value threshold of results displayed). Database searches include a variety of vertebrate genomes (see Figure 3) as well as transcribed sequence databases such as EMBL EST and Unigene and ncRNA databases that are updated on a regular basis. This extensive resource has a number of uses, predominant of which is identifying those genomes in which the CNE has been duplicated in lineage (such as those at the origin of vertebrates [31] or within the teleosts [33]) or species-specific duplication events.

• To aid evolutionary analyses of CNEs, and utilising the BLAST results, a user can choose any combination of vertebrate genome from which to produce a multiple alignment of the CNE (from a choice of two aligners) allowing lineage or species specific mutational events to be detected. The output produces the multiple alignment in a number of formats (such as FASTA, MSF etc) that can be directly viewed in the browser or downloaded for further downstream analysis.

• CNEs from each of the main genomes can be downloaded in bulk as a set of sequences in FASTA format or as a tab-delimited file with substantial sequence and positional data. Bulk downloads can also be filtered by genome, gene region or CNE length and conservation.

### Technical specifications

The database is run on a Solaris platform and housed using the MySQL (v1.4.0) database management system at the School of Biological and Chemical Sciences, QMUL, London. The web interface is run on an Apache web server that serves a set of CGI scripts and static web pages. The "CONDOR-View" graphical browser is created using the Bioperl Bio::Graphics module [35]. CNEs are served to the Ensembl Genome browser using the ProServer DAS server (v2) [36].

## Conclusion

The identification and characterisation of distal CRMs that control the complex gene regulatory networks in early vertebrate development remain one of the greatest challenges of the post-genomic era. It is of increasing interest to the scientific community for datasets of predicted CRMs that are well defined, easily accessible and manageable in number. Here we present CONDOR, a database resource of both predicted and experimentally verified orthologous non-coding elements conserved across the vertebrate lineage and associated with early developmental regulators. This resource is designed both for the increasing number of experimental groups interested in prioritising a set of elements for experimental verification and computational users interested in training sets with high regulatory potential.

## Availability and requirements

The CONDOR database is publicly available without payment or registration at . A full tutorial can be found on the website. Please cite this paper when publishing data using computational data from CONDOR. Use of functional data in publications is only permitted with prior agreement from the corresponding author. Positions of CNEs within vertebrate genome assemblies are constantly updated as new versions are released and *in vivo *zebrafish enhancer annotations are added to the database automatically once results for each experiment are collated.

## Authors' contributions

AW carried out all multiple alignments, computational data extraction and post-processing, designed and implemented the database schema and web front end and wrote the first draft of the manuscript. JC designed the zebrafish expression sub-domains and colour-coding system involved in the *in vivo *data collection. DG, JC, SS, PS, HC and GE developed and carried out the *in vivo *enhancer assays. DG and GM helped write parts of the manuscript. GE conceived the database, and participated in its design and coordination.
